# Prevailing practices in the use of antibiotics by dairy farmers in Eastern Haryana region of India

**DOI:** 10.14202/vetworld.2018.274-280

**Published:** 2018-03-04

**Authors:** Vikash Kumar, Jancy Gupta

**Affiliations:** Division of Dairy Extension, National Dairy Research Institute, Karnal, Haryana, India

**Keywords:** antibiotic usage, treatment schedules, veterinary consultancy, withdrawal period

## Abstract

**Aim:**

The aim of the study was to assess the antibiotic use in dairy animals and to trace its usage pattern among the small, medium, and large dairy farmers in Eastern Haryana region of India.

**Materials and Methods:**

Karnal and Kurukshetra districts from Eastern region of Haryana state were purposively selected, and four villages from each district were selected randomly. From each village, 21 farmers were selected using stratified random sampling by categorizing into small, medium, and large farmers constituting a total of 168 farmers as respondents. An antibiotic usage index (AUI) was developed to assess usage of antibiotics by dairy farmers.

**Results:**

Frequency of veterinary consultancy was high among large dairy farmers, and they mostly preferred veterinarians over para-veterinarians for treatment of dairy animals. Small farmers demanded low-cost antibiotics from veterinarians whereas large farmers rarely went for it. Antibiotics were used maximum for therapeutic purposes by all categories of farmers. Completion of treatment schedules and follow-up were strictly practiced by the majority of large farmers. AUI revealed that large farmers were more consistent on decision-making about prudent use of antibiotics. Routine use of antibiotics after parturition to prevent disease and sale of milk without adhering to withdrawal period was responsible for aggravating the antibiotic resistance. The extent of antibiotic use by small farmers depended on the severity of disease. The large farmers opted for the prophylactic use of antibiotics at the herd level.

**Conclusion:**

Antibiotic usage practices were judicious among large dairy farmers, moderately prudent by medium dairy farmers and faulty by small farmers. The frequency of veterinary consultancy promoted better veterinary-client relationship among large farmers.

## Introduction

Antibiotics can be regarded as an “endangered species” facing extinction due to the emergence of antibiotic resistance and void in the continuous development of new antibiotics [1]. The extensive and inappropriate use of antibiotics in animal production and dairy farming has led to a global rise in multi-resistant microbes which are spreading rapidly and is not confined to developing countries [2]. The regular clinical inspection of dairy animals and use of antibiotic prescription based on sensitivity testing should be promoted to reduce the over-use of antibiotics [3]. Lack of proper data related to antimicrobials drug use in India limits the understanding of type and magnitude of antibiotic usage in dairy animals [4]. The therapeutic and prophylactic purposes have validated benefits such as improved animal health and increase in production levels, but it increases the pace of antibiotic resistance [5]. The sub-therapeutic use of antibiotics has been considered as a driver for the emergence of antibiotic resistance in countries where it is used for growth promotion [6].

Judicious prescription and antibiotic conservation practices of veterinarians are affected by the demand of farmers to get antibiotics by giving reference to past treatment leads [7]. The strategy for prudent use among stakeholders needs to be more practical and integrated. Tracing the antibiotic residues in milk at the milk procurement site can aid policymakers, organizations and animal health-care professionals to ensure availability of quality milk to consumers [8]. Since dry cow therapy promotes the use of antibiotic routinely for the prophylactic purpose, it accelerates antibiotic resistance [9]. The voluntary ban of dry cow therapy has led to a 92% reduction in the use of antibiotics between 2009 and 2012 in the Dutch countries [10].

Limited research has been done to assess antibiotic use in milk production in India. So in the present study, an antibiotic usage index (AUI) was developed to measure antibiotic usage pattern of dairy farmers.

## Materials and Methods

### Ethical approval

No animal was used for the study. Hence, ethical approval was not needed.

### Research design

This study was a non-experimental research design.

### Study site

The study was conducted in Eastern region of Haryana state of India. Haryana ranks second in the country in per capita per day availability of milk. The Eastern Haryana region has 1157 veterinary institutions and has 53.3 and 58.3% of total cow and buffalo population of the state, respectively. Karnal and Kurukshetra districts of Haryana were purposively selected, and four villages from these districts were randomly selected. The study was conducted during 2016-2017.

### Data collection

From each village, 21 farmers were selected randomly using stratified random samplings by categorizing into small, medium, and large farmers constituting a total of 168 farmers as respondents. The farmers rearing one to four animals were categorized as smallholder dairy farmers, four to 10 animals as medium holder dairy farmers and those with more than 10 animals were considered as large dairy farmers for the purpose of this study following relevant literature [11].

### Procedures

#### Preparation of AUI

An AUI was developed by incorporating 19 indicators under four dimensions of the use of antibiotics, i.e., symptom level, cow level, herd level, and perceived alternatives, based on the opinion of a panel of 40 experts. Under symptom level indicators were frequency of veterinary consultancy, experience of treatment, seeking therapeutic intervention, alteration in dose of antibiotics, and change in mode of administration of antibiotics. Contingent use of antibiotics, varying the dose of antibiotics according to severity of disease, sale of milk of treated animal, discontinuance of treatment after the disappearance of symptoms and increase in dose in case of poor response to treatment were indicators of animal level usage of antibiotics. Routine use of antibiotics, animal husbandry practices, dry cow therapy and preference to animals according to milk quota in diagnosis were the indicators under the herd-level dimension of antibiotic usage. Four indicators, namely, vaccination, physical barriers such as teat sealant, indigenous traditional knowledge, and use of prebiotics/probiotics to prevent animal disease were selected under the perceived dimensions of alternatives to the antibiotics in the study. For making the indicators scale-free, following methods were applied:


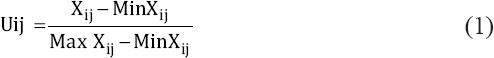



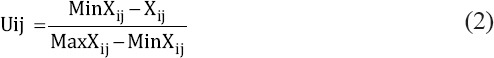


Where,

i=1, 2, 3,…, n indicators

j=1, 2, 3 dimensions of judicious use of antibiotics

X_ij_=Value of i_th_ indicator of j^th^ dimension.

Equation (1) was applicable for indicators having positive implications on the use of antibiotics. Equation (2) was applicable for indicators having negative implications on judicious use of antibiotics.

The composite AUI for farmers was calculated by taking the weighted mean of three dimensions, i.e.,


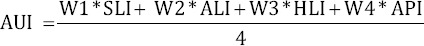


Where,

W=Weight assigned to respective dimensions

SLI=Symptom level index

ALI=Animal level index

HLI=Herd level index

API=Alternatives perceived index

AUI=Antibiotic usage index.

The relevancy weightage (RW) and mean relevancy score (MRS) were worked out. The indicators with statements having RW >0.70 and MRS >2.25 were considered for including in the usage index.

AUI was developed, and score of each dimension ranged from 0 to 1. The responses of farmers were recorded on this AUI which reflected the degree of judicious decision-making regarding antibiotic use.

### Statistical analysis

AUI was prepared by expert judgment method. Cumulative square root frequency was used for categorization of farmers according to the antibiotic usage pattern.

## Results

### Sources of consultancy of farmers

The veterinary consultancy including the frequency of contact with veterinarians was found low among small farmers (50.00%) in comparison to medium farmers (76.79%) and large farmers (87.50%). It is clear from Table-1 that 50% of small farmers consulted veterinarians, 30.36% of them consulted paravets, 12.50% of them procured antibiotics through over-the-counter sales, and 7.14% of them obtained it through milk vendors on the occurrence of disease on a regular basis. The consultancy with paravets, milk vendors and over-the-counter sales for obtaining antibiotics were prevalent among small farmers.

**Table-1:** Distribution of farmers as per sources of veterinary consultancy (n=168).

Consultancy sources	Small farmers (n=56)	Medium farmers (n=56)	Large farmers (n=56)
Veterinarians	50.00	76.79	87.50
Paravets	30.36	17.85	12.50
Over-the-counter sales	12.50	3.57	0
Milk vendors	7.14	1.79	0

Numerical figures indicate %

### Veterinary consultancy services utilized by farmers

The utilization of veterinary services and its frequency are enlisted in Table-2. A perusal of the Table-2 shows that 32.15% of small farmers and 89.29% of large farmer opted vaccination as a precautionary measure, and consultancy with veterinarians was having a direct relation with the frequency of use of vaccination. Similarly, the consultancy by veterinarians for disease treatment was found 62.57, 75.00, and 91.07% by small, medium, and large farmers, respectively. Deworming (75.00%) and dry cow therapy (78.58%) were practiced by large farmers more frequently than small and medium farmers of the study area.

**Table-2:** Distribution of farmers as per veterinary consultancy services utilized by them (n=168).

Services utilized	Small farmers (n=56)			Medium farmers (n=56)			Large farmers (n=56)		
		
	Never	Sometimes	Always	Never	Sometimes	Always	Never	Sometimes	Always
Vaccination	8.93	58.92	32.15	3.57	26.79	69.64	0	10.71	89.29
Completion of disease treatment	19.64	17.86	62.57	0	25.00	75.00	0	8.93	91.07
Deworming	41.07	30.36	28.57	19.64	35.71	44.65	7.14	17.86	75.00
Dry cow therapy	78.58	14.28	7.14	23.21	25.00	51.79	8.92	12.50	78.58

Numerical figures indicate %

### Alternatives for disease treatment as perceived by large farmers

Small farmers frequently used alternatives such as indigenous technical knowledge (12.51%), homeopathic (5.36%), and ayurvedic medicines (3.57%) in animal husbandry. It was observed that large farmers hardly relied on indigenous technical knowledge as well as ayurvedic and homeopathic medicines. (Table-3).

**Table-3:** Distribution of respondents as per alternatives for allopathic medicines as perceived by them (n=168).

Services utilized	Small farmers (n=56)			Medium farmers (n=56)			Large farmers (n=56)		
		
	Never	Sometimes	Always	Never	Sometimes	Always	Never	Sometimes	Always
Indigenous technical knowledge	69.64	17.85	12.51	92.86	5.36	1.78	96.42	2.79	0.90
Ayurvedic medicines	80.36	14.29	5.36	89.29	5.36	5.35	92.86	5.36	1.78
Homeopathic medicines	89.29	8.93	3.57	92.86	3.57	1.78	96.42	2.79	0.90

Numerical figures indicate %

### Purposes of antibiotic use by farmers

It was found that majority of small farmers (98.21%) used the antibiotics for therapeutic purpose, while it is subtherapeutic use was found very low (1.79%) (Table-4). Medium farmers availed antibiotic use for therapeutic purpose (83.92%), subtherapeutic purpose (8.93%), and prophylactic purpose (7.15%). It was interesting to note that large farmers used antibiotic for therapeutic purpose (73.21%) and subtherapeutic purpose (8.93%) and also relied on other choices for prophylactic use of antibiotics (17.86%).

**Table-4:** Distribution of respondents according to purpose of antibiotic use (n=168).

Purpose	Small farmers (n=56)	Medium farmers (n=56)	Large farmers (n=56)
Therapeutic purpose	98.21	83.92	73.21
Subtherapeutic purpose	1.79	8.93	8.93
Prophylactic purpose	0	7.15	17.86

Numerical figures indicate %

On the basis of cumulative square root frequency, the three categories (low, medium, and high) were used to locate the farmers according to the appropriateness of decision-making. Table-5 indicates the decision-making at all four levels (symptom, cow, herd, and alternatives perceived), irrespective of suitability of particular level of decision-making to a particular category of farmer (small, medium, and high).

**Table-5:** Distribution of dairy farmers with respect to antibiotic usage practices.

Dimensions	Small farmers	Medium farmers	Large farmers
Symptom level	0.18	0.04	0.01
Animal level	0.09	0.22	0.18
Herd level	0.06	0.13	0.28
Alternatives perceived	0.01	0.02	0.05

Continuum: 0-1

After considering the overall antibiotic usage practices by small, medium, and large farmers it was found that large farmers were more consistent with the prudent use of antibiotics as reflected from the score of AUI (Table-5).

Table-6 reveals that 46.44% of small farmers were least considerate (<0.07 score on AUI) to antibiotic resistance as revealed in usage pattern of antibiotics. The score for judicious use of antibiotic was low (score of <0.14 on AUI) among 59.29% medium farmers in their level of appropriateness of decision-making. However, 46.42% large farmers fell in medium category (score of 0.26-0.34 on AUI) of judicious use of antibiotics.

**Table-6:** Distribution of respondents as per usage pattern of antibiotics (n=168).

Category	Frequency n=56 (%)
Small farmers (n=56)	
Low (<0.07)	26 (46.44)
Medium (0.090.15)	22 (39.28)
High (>0.15)	8 (14.28)
Medium farmers (n=56)	
Low (<0.14)	36 (59.29)
Medium (0.140.20)	17 (30.35)
High (>0.20)	3 (10.36)
Large farmers (n=56)	
Low (<0.18)	20 (35.71)
Medium (0.260.34)	26 (46.42)
High (>0.26)	10 (17.85)

Continuum: 0-1

The respondents were classified into three categories (low, medium, and high) according to cumulative square root frequency. Table-7 reveals that 39.28% of small farmers were least considerate (<0.11 score on AUI) to antibiotic resistance at symptom level of decision-making to use antibiotics. The consideration to judicious use of antibiotic was low (score of <0.19 on AUI) among 59.29% medium farmers at an animal level to go for better antibiotic use practices. However, 38.60% large farmers fell in medium category (score of 0.24-0.30 on AUI) of judicious use of antibiotics at the herd level.

**Table-7:** Distribution of farmers as per level of decision-making among different farmer categories (n=168).

Category	Frequency n=56 (%)
Decision-making at symptom level by small farmers (n=56)	
Low (<0.11)	23 (39.28)
Medium (0.11-0.19)	20 (35.71)
High (>0.19)	13 (23.21)
Decision-making at cow level by medium farmers (n=56)	
Low (<0.19)	27 (47.55)
Medium (0.19-0.24)	24 (41.38)
High (>0.24)	6 (11.07)
Decision-making at herd level by large farmers (n=56)	
Low (<0.24)	18 (33.33)
Medium (0.24-0.30)	22 (38.60)
High (>0.30)	16 (28.07)

Continuum: 0-1

Levels of decision-making regarding the antibiotic usage were observed under the following subheads.

### Symptom level

The decisions of smallholder dairy farmers regarding the antibiotic use were mainly focused at symptom level because of small herd size and resultant ease of observation (Table-5). It was revealed through usage index score of 0.18 (Table-6).

### Animal level

Majority of the medium farmers made the decision regarding the antibiotic use at animal level as revealed through usage index score of 0.22 (Table-6).

### Herd level

Majority of large farmers adhered to the decision regarding antibiotic use at herd level. The usage index score of 0.28 among large farmers was more consistent than small farmers (0.06) and large farmers (0.13) at herd level. Furthermore, the consistency and accuracy of level of decision-making in the respective categories increased from small farmers (0.18) to the medium farmers (0.22) and highest for large farmers (0.28) at symptom, cow, and herd level, respectively (Table-6).

### Alternatives perceived

The usage index score of 0.05 for utilizing or seeking to utilize alternatives with respect to large farmers was more judicious than medium farmers (0.03) and small farmers (0.01).

## Discussion

Over-the-counter sales of antibiotics (either without a prescription or by reusing old prescriptions) and informal consent with paravets and veterinarians ensured high antibiotic consumption in the farms [12]. There were no limitations in choice to obtain antibiotics, i.e., over-the-counter, veterinary-client relationship or a written prescription from a veterinarian. The sources of obtaining antibiotics were subjected to change according to the type and severity of the disease in the study area. The smallholder dairy farmers in the study area chose to get antibiotics as suggested by milk vendors or dairy cooperatives because they perceived that these sources as credible and tried to escape the financial burden of veterinary consultancy. The farmers wished to fulfill their social expectations and preference than to obey medical or scientific rational by the veterinarians. Similar findings regarding the social expectations and prescription were reported by Paredes *et al*. [13].

This study revealed that frequency of disease and choice of primary antibiotics for disease treatment were influenced by herd size. Increasing herd size has been associated with increased morbidity and mortality if antibiotics were not used [14]. Monitoring the antibiotic programs, adherence to its guidelines to promote prudent use and educational campaigns could be helpful to minimize the spread of antimicrobial resistance [15]. Regular vaccination was practiced among 32.15% small farmers and 69.64% of medium farmers (Table-2). Similar findings of low vaccination among farmers were reported by Rathod *et al*. [16]. According to Rathod *et al*. [17], benefits of vaccination should be made available through campaign and extensive livestock extension activities to educate them about its ability to control economic losses due to disease.

Scanty information is present about antibiotic use in low-income countries like India. As pointed by Redding *et al*. [18], understanding of pattern and reason for the use of antibiotics is needed to promote its judicious use. Antibiotics were used for therapeutic purpose when the animal had reached an advanced stage of clinical disease. The use of antimicrobial drugs as both growth promoters and disease prevention, at subtherapeutic doses were reported by McEwen and Fedorka-Cray [19]. The present study indicated the wide use of antibiotics for therapeutic purpose in contradiction to the findings of National Research Council of USA [20] that most of the antibiotics in animal agriculture were used as growth promoters or prophylactic purpose. In India, few regulations exist against the nontherapeutic use of antibiotics with no rigorous implementation protocols. As a result, overuse of antibiotic in animal sector had aggravated the resistance [21]. Thus, the therapeutic regimens of the drug may increase this risk of antibiotic resistance [22]. The findings of the present study were consistent with the reporting of Sahoo *et al*. [23] that non-completion of scheduled treatment in India was due to lack of financial resources among smallholder dairy farmers (Table-2). It was also observed that due to lower educational levels the knowledge of farmers regarding appropriate use of antibiotics was very less and often limited [24]. It was observed that few farmers were using those antibiotics which were the second choice of the veterinarians because of its cost-effectiveness.

Majority of small farmers opted for therapeutic use of antibiotics because they consulted veterinarians after manifestations of symptoms in the advanced stage of disease (Table-4). Medium level farmers went for therapeutic as well as subtherapeutic use of antibiotics for healthcare compared to small farmers. Large farmers availed all the options of the purpose of antibiotic use as stated earlier, because of their large herd size and good economic condition. Small farmers did not make decision at herd level, so the prophylactic use was absent. Prophylactic use was found among large farmers because they preferred it for indirect herd protection and made the decision at herd level. In Eastern Haryana, smallholder farmers use antibiotics for treatment of sick animals rather than growth promotion or disease prevention (Table-4). Furthermore, concerns about profitability in dairy farming induce them to use relatively lesser amounts of antibiotics because they often operate on narrow margins. Large farmers frequently use antibiotics because animals are kept in a high density of herd [24].

Higher levels of education among large farmers led to higher levels of awareness and knowledge about antibiotic resistance [25]. For improving animal health and welfare, the over-dependence on antibiotics need to be reduced. The use of vaccines could pave the way for antibiotic conservation and significantly reduce antibiotic consumption [5]. The decision-making of small farmers was at symptom level, medium farmers at cow level, and large farmers at herd level [26]. Most of farmers were unaware about the harmful effects of certain types of antibiotics and they considered only efficacy of medicine to cure the disease the larger farmers revealed that the advice of veterinarian on suboptimal use of antibiotics and its relation to antibiotic resistance was lacking and these findings were consistent with the reporting of Vaarst *et al*. [27]. The selective pressure due to use of antimicrobials in food-producing animals (including dairy animals) led to emergence of horizontal transfer of antimicrobial-resistant determinants in bacteria [28]. The overuse and inappropriate use of antibiotics could be countered through antibiotic stewardship programs. The package of practices should aim at judicious use of antibiotics accompanied with monitoring and evaluation to promote antibiotic conservation practices [29].

### Decision-making regarding use of antibiotics at symptom level

The usage index score of 0.18 was found for smallholder dairy farmers regarding usage pattern of antibiotics because the frequency of veterinary consultancy was affected by severity of disease, observed symptom, and familiarity with disease (Table-5). This score was found more consistent to medium farmers than small farmers (0.09) and large farmers (0.18) at this level. Few small farmers preferred treating animals with previous experience, previous prescriptions and based on information from feed store, training, peers, etc. The small farmers seek therapeutic intervention only when milk yield declined and manifestation of symptom in advance stage of disease. Increase in a dose of antibiotic according to the severity of disease without veterinary consultation accounted for the low efficacy of decision regarding the use of antibiotics (Table-5). The similar findings were reported by Dunn-Rankin and King [30] that small farmers opted for antibiotic treatment by assessing symptoms followed by administration of antibiotics.

### Decision-making regarding use of antibiotics at animal level

The usage index score of antibiotics 0.22 for medium farmers was more judicious than small farmers (0.18) (Table-5). The consistency and accuracy of decision-making in their categories increased from small farmers (0.18) to the medium farmers (0.22) in the succeeding level. The contingent use residual antibiotics, its routine use after parturition, calf-feeding to animal under treatment, and sale of its milk without consideration of the withdrawal period were responsible for the unresponsive decision and injudicious use of antibiotics. The milk of antibiotic-treated animal could be made suitable for calves after pasteurization and electrochemical oxidation of raw milk with oxytetracycline at 100 mg/mL [31]. Most of the farmers discontinued the treatment when diseases seem to be cured due to the absence of symptoms. They increased the dose in case of poor response and treatment failure.

### Decision-making regarding use of antibiotics at herd level

The large farmers were found to use antibiotics more judiciously than small and medium dairy farmers. The antibiotics usage index score of 0.28 for large farmers revealed more prudent use of antibiotics than small farmers (0.18) and medium farmers (0.22) (Table-5). The special preference to high milk yielding animals in relation to milk quota biased the treatment to them and advocated the dry cow therapy among large farmers. Oliver *et al*. [4] reported that over 90% of dairy farms practiced antibiotic dry cow therapies in developed countries. Their reporting was contradicted in the present study area where farmers practicing dry cow therapy as regular practice were even <50% (Table-2). Surprisingly, it was observed that milk of antibiotics treated animals were fed to calf and also sold by all the respondents. The prophylactic use of antibiotic by large farmers was prevalent in the study area. The veterinarians and paravets prescribed prophylactic drugs mainly to prevent foot and mouth disease and control mastitis at herd level. Large farmers revealed that reducing the use of antibiotics would lead to economic loss and unfulfilled production goal to farmers. The use of antibiotics can be estimated by counting the number of days per year the average cow in a herd receives antibiotic treatment [32]. It was found that dry cow therapy and clinical mastitis accounted for maximum doses of antibiotics. It was observed that the farmers in the study area rarely screen herds for endemic bacteria. Hence, tangible data were not available about the prevalence of resistance in these bacteria [5].

### Alternatives perceived to reduce use of antibiotics

Use of teat sealant during critical time of dry period, looking forward toward use of homeopathy and indigenous technical knowledge, use of probiotics/prebiotics could be alternatives perceived by the farmers. The usage index score of 0.05 was found for large farmers which were more judicious than medium farmers (0.03) and small farmers (0.01) for the alternatives of antibiotics (Table-5). Organizations for economic cooperation and development countries are trying to reduce antibiotic use in livestock through new policies and interventions [10]. Nair *et al*. [33] reported that ayurvedic remedies reduced the number of antibiotic positive milk samples by 18-49% in India. Nano-antibiotics could be priority over generic drugs and broad-spectrum antibiotics to achieve cost-effectiveness [34]. The Red Line Campaign (2016) and The National Antimicrobial Resistance Containment Policy (2011) recommended to establish a separate schedule H1 under the drugs and cosmetics rules, to legalize the sales and color-coded tagging of antibiotics and reduce the usage of antibiotics in livestock animals in India [7].

## Conclusion

Irrational use of antibiotics in dairy farming in Eastern Haryana are aggravated by poor knowledge and misconceptions about antibiotics, false practices, limited supervision, and easy access to antibiotics. It demands prompt action on antibiotic misuse coupled with continuing education and counseling for veterinarian about prudent use of antibiotics. Farmers with good economic condition utilized better veterinary consultancy services and performed best antibiotic usage practices. For reducing the pace of antibiotic resistance, no single approach can solve the problem because of interwoven complex interests of all stakeholders. Cumulative impact of numerous interventions will have a significant impact if practiced in well-organized manner followed by development of new antibiotics. Formulating new policies on antibiotic resistance are not likely to be transformative unless role of all the stakeholders involved in decision-making regarding the antibiotic use are taken into account. A contingent system approach by integrating the farmers, veterinarians, and other stakeholders offering robust antibiotic stewardship along with a properly designed antibiotic resistance surveillance program is required.

### Authors’ Contributions

JG designed the research work and VK and JG executed the work at field level. VK analyzed the data. Both authors contributed equally in preparation and revision of the manuscript. All authors read and approved the final manuscript.
